# Impact of Fascial Orientation of the Radial Artery Graft on Mid-Term Patency and Clinical Outcome

**DOI:** 10.3390/jcm15145489

**Published:** 2026-07-13

**Authors:** Yoganiranjana Dharuman, Samir Sirat, Mirko Doss

**Affiliations:** 1 Department of Cardiothoracic Surgery, University Hospital Aachen, Pauwelsstraße 30, 52074 Aachen, Germany; 2 Department of Cardiothoracic Surgery, Helios Hospital Siegburg, Ringstraße 49, 53721 Siegburg, Germany

**Keywords:** radial artery, coronary artery bypass grafting, graft patency, fascial orientation, graft kinking, coronary revascularization

## Abstract

**Background:** The radial artery (RA) is widely used as a conduit in coronary artery bypass grafting. Its fascial layer provides mechanical stability but may reduce graft flexibility and promote kinking when routed over the margo acutus during right coronary artery bypass. This study investigated the impact of graft fascial orientation on RA patency and clinical outcomes. **Methods:** In this retrospective observational cohort study with prospective follow-up, 100 patients undergoing CABG with the RA grafted to the right coronary artery were included. Patients were assigned to a “prone” group (*n* = 50), with the fascial side oriented toward the sternum, or a “supine” group (*n* = 50), with the fascia facing the epicardium. Clinical follow-up was performed after a mean of 4.25 years. MDCT-based graft patency assessment was available in 89 patients. **Results:** Overall survival at follow-up was 100%, with no postoperative myocardial infarctions or reoperations. Overall RA patency was 94%, with patency rates of 91.5% in the supine group and 97.6% in the prone group (*p* = 0.37). Internal mammary artery and saphenous vein graft patency rates were 92.8% and 96.4%, respectively. Competitive flow showed a near-significant association with RA occlusion (*p* = 0.09). **Conclusions:** RA grafts demonstrated excellent mid-term patency. Fascial orientation of the pedicled RA graft was not significantly associated with graft patency.

## 1. Introduction

Coronary artery bypass grafting (CABG) remains one of the most effective surgical strategies for the treatment of advanced coronary artery disease. While the left internal mammary artery (LIMA) has been considered the gold standard conduit for myocardial revascularization because of its excellent long-term patency, the search for additional durable arterial grafts has led to the reintroduction of the radial artery (RA) into clinical practice since the early 1990s. Owing to its favorable anatomical location, appropriate vessel caliber, and sufficient graft length, the RA has become an increasingly popular conduit for achieving complete arterial revascularization. Current evidence and international expert reviews support the RA as the preferred second arterial conduit because of its superior long-term patency compared with saphenous vein grafts [1].

Numerous clinical studies have demonstrated excellent short- and long-term outcomes of RA grafting, with patency rates approaching those of internal mammary artery grafts. Compared with saphenous vein grafts, the RA has shown superior long-term durability and improved clinical outcomes, including lower mortality rates and reduced graft failure [2]. Furthermore, advances in harvesting techniques and perioperative vasodilatory management have significantly reduced the incidence of vasospasm, once considered the principal limitation of the RA conduit.

Despite these favorable results, several studies have reported comparatively lower patency rates when the RA is used for revascularization of the right coronary artery (RCA). Various mechanisms have been proposed to explain this phenomenon, including competitive flow due to moderate native coronary stenosis, the greater graft length required for distal RCA anastomoses, and mechanical stress caused by routing the graft around the margo acutus of the heart. However, the influence of graft orientation and the anatomical relationship between the fascial side of the RA pedicle and the heart has not been systematically investigated.

The pedicled harvesting technique preserves the accompanying veins, perivascular tissue, and fascial structures surrounding the RA, thereby improving graft stability and potentially protecting against vasospasm [3]. At the same time, these structures may reduce graft flexibility and contribute to kinking when the graft is exposed to torsion or excessive tension, particularly in distal RCA bypass configurations.

The present study aimed to evaluate whether the orientation of the fascial side of the radial artery graft influences graft patency and clinical outcomes after CABG. Specifically, two different graft orientations (“prone” versus “supine”) were compared in patients undergoing RCA bypass grafting with the RA, using postoperative multidetector computed tomography (MDCT) follow-up and mid-term clinical assessment.

### Research Aim

The radial artery, together with its accompanying venous structures and surrounding fascial tissue, provides structural support to the graft pedicle after harvesting. While this anatomical configuration contributes to mechanical stability, it may also reduce the effective usable length of the conduit. During coronary artery bypass grafting, the positioning of the graft, particularly when the fascial layer is oriented outward, and the vessel is routed around the margo acutus of the heart, can lead to geometric distortion of the conduit. This may result in kinking of the graft, with the phenomenon being especially relevant in bypass procedures of the right coronary artery, where the course of the graft is more prone to sharp angulation.

The aim of this study was to investigate the influence of fascial orientation relative to the heart on mid-term graft patency and clinical outcomes after radial artery bypass grafting to the right coronary artery.

## 2. Methods

This study was designed as a retrospective, non-randomized observational cohort study with prospective clinical and radiological follow-up. The retrospective component comprised the analysis of perioperative and baseline data from patients undergoing coronary artery bypass grafting (CABG) using the radial artery (RA) as a pedicled graft to the right coronary artery (RCA). The prospective component consisted of a standardized clinical follow-up interview and multidetector computed tomography (MDCT) assessment of graft patency.

Patients were identified from two institutional surgical databases comprising a total of 521 patients who had undergone CABG with use of the radial artery during the study period. From this source population, 100 symptomatic patients fulfilling the predefined inclusion and exclusion criteria were included in the present analysis. The radial artery was evaluated preoperatively by ultrasound. Before graft implantation, the harvested radial artery was routinely probed intraoperatively with a 1.5 mm coronary probe to confirm an adequate luminal diameter and graft quality. The study cohort was divided into two groups according to the intraoperative orientation of the pedicled RA graft actually used during surgery: a “supine” group, in which the fascial side of the graft faced the epicardium, and a “prone” group, in which the fascial side was oriented toward the sternum. During the study period, graft orientation reflected the standardized surgical technique of the operating surgeon. One surgeon consistently used the prone orientation, whereas the other consistently used the supine orientation. Clinical follow-up was performed using a standardized questionnaire, and graft patency was assessed by 64-slice multidetector computed tomography (MDCT). The mean follow-up duration was 4.25 years.

### 2.1. Study Population

Patients were identified from two institutional databases comprising 521 patients who had undergone CABG using the radial artery as a conduit. Only patients who underwent radial artery grafting to the right coronary artery and completed symptomatic follow-up were eligible for inclusion. After application of the predefined inclusion and exclusion criteria, 100 patients were included in the final study cohort (50 in the “supine” group and 50 in the “prone” group).

The retrospective study was not randomized, and no propensity score matching was performed. Patients were allocated according to the documented intraoperative orientation of the radial artery graft.

At follow-up, MDCT assessment of graft patency was available in 89 of the 100 patients. Three patients in the “supine” group and eight patients in the “prone” group did not undergo MDCT because of missing imaging data or logistical reasons but remained part of the clinical follow-up analysis.

### 2.2. Inclusion Criteria

The following inclusion criteria were applied:

- Presence of left main coronary artery disease or multivessel coronary artery disease requiring primary CABG.
- Left ventricular ejection fraction ≥ 35%.
- Significant stenosis of the right coronary artery (RCA), generally ≥ 70%.
- Preoperative ultrasound demonstrating an adequate radial artery diameter for use as a bypass conduit, with intraoperative confirmation using a 1.5 mm coronary probe.
- Use of the RA as a pedicled graft anastomosed to the RCA.
- Availability of operative documentation allowing assignment of graft orientation to either the “supine” or “prone” configuration.

### 2.3. Exclusion Criteria

The following exclusion criteria were applied:

- Emergency CABG procedures.
- Renal insufficiency (serum creatinine > 1.7 mg/dL) because of the contraindication to contrast-enhanced MDCT imaging.
- Known allergy to iodinated contrast media.
- Insufficient operative documentation preventing reliable classification of the radial artery graft orientation.
- Previous cardiac surgery.
- Patients who declined participation in the clinical and radiological follow-up.
- Patients without available MDCT imaging at follow-up were excluded from the graft patency analysis but remained part of the clinical follow-up cohort.

### 2.4. Surgical Technique of Coronary Artery Bypass Grafting

All 100 patients underwent surgery under general anesthesia with cardiopulmonary bypass support. Following median sternotomy, opening of the pericardium, and initiation of extracorporeal circulation, the coronary bypass grafts were performed under cardioplegic cardiac arrest. Cardiac arrest was induced by aortic cross-clamping and administration of warm Calafiore cardioplegic solution.

Depending on the individual coronary anatomy and the distribution of stenotic lesions, complete myocardial revascularization was achieved using multiple bypass grafts. In all patients, the radial artery (RA) was used as a graft and anastomosed to the right coronary artery (RCA). The right internal mammary artery (RIMA), left internal mammary artery (LIMA), and/or great saphenous vein (GSV) were additionally used as conduits for revascularization of other coronary vessels, as individually required.

The radial artery was harvested from the non-dominant arm according to the Reyes technique. In the “prone” group, the graft was anastomosed to the heart with the fascia oriented toward the sternum, whereas in the “supine” group, the anastomosis was performed with the fascia oriented toward the epicardium. All anastomoses were performed using a continuous 7-0 polypropylene suture technique.

After completion of revascularization of all stenotic coronary vessels and achievement of adequate hemostasis, the sternum was closed using titanium wires, followed by standard skin closure.

### 2.5. Patient Follow-Up

Follow-up consisted of a structured clinical assessment and radiological evaluation of graft patency using multidetector computed tomography (MDCT).

### 2.6. Clinical Follow-Up

Clinical follow-up was performed at a mean of 4.25 years after surgery using a standardized telephone-based questionnaire comprising 18 predefined items. The questionnaire assessed the postoperative cardiovascular course, including the occurrence of myocardial infarction, repeat coronary interventions, recurrent angina pectoris, and dyspnea. In addition, relevant cardiovascular risk factors, including diabetes mellitus, arterial hypertension, renal insufficiency, hypercholesterolemia, and smoking status, were documented. All 100 patients participated in the clinical follow-up analysis.

### 2.7. Radiological Follow-Up Examination

All 100 patients were scheduled for 64-slice multidetector computed tomography (MDCT) at the outpatient radiology department of our institution. Prior to the examination, each patient was informed by the examining physician about the indication, procedure, and potential risks of contrast-enhanced MDCT imaging. Written informed consent was obtained before the examination. Scanning was performed by two radiology residents. Subsequently, the coronary arteries and bypass grafts were evaluated, and the final report was reviewed and, if necessary, corrected by a senior radiologist.

MDCT-based graft patency assessment was available in 89 patients. Patients without evaluable MDCT imaging were excluded from the graft patency analysis but remained part of the clinical follow-up cohort.

On the day of examination, all patients were required to present a serum creatinine value obtained within the previous 3 weeks.

## 3. Data Analysis

Data analysis was based on preoperative, intraoperative, and postoperative variables collected from both study groups (Table 1, Table 2 and Table 3). Statistical analyses were performed using the software package BiAS (Version 10. epsilon-Verlag, Hofheim, Germany) (Biometric Analysis of Samples).

Continuous variables are presented as mean ± standard deviation (SD), whereas categorical variables are presented as absolute numbers and percentages. Comparisons of categorical variables between the two study groups were performed using Fisher’s exact test. Overall survival was analyzed using the Kaplan–Meier method. A two-sided *p*-value < 0.05 was considered statistically significant.

## 4. Results

### 4.1. Demographic Data

Baseline demographic and clinical characteristics of the study cohort are summarized in Table 4. The study population consisted predominantly of male patients in both groups. In the “supine” group, 92% (*n* = 46) of patients were male, compared to 78% (*n* = 39) in the “prone” group. Accordingly, 8% (*n* = 4) of patients in the “supine” group and 22% (*n* = 11) in the “prone” group were female.

Mean age and body mass index were similar between the groups. The mean age ranged between 61 ± 10 years in one group and 64 ± 8.28 years in the other group.

The data show a mean age of 64.16 years and a mean body mass index (BMI) of 28.01 kg/m^2^ in the “supine” group.

The data show a mean age of 62.16 years and a mean body mass index (BMI) of 28.58 kg/m^2^ in the “prone” group.

With regard to medical history and cardiovascular risk profile, most baseline variables did not differ significantly between the “supine” and “prone” groups. However, peripheral arterial occlusive disease was significantly more frequent in the prone group and therefore represents an important baseline imbalance that should be considered when interpreting the follow-up results.

### 4.2. Preoperative Medical History Data

The medical history data are presented in Table 5. In contrast to the “supine” group, the “prone” group showed a significantly higher proportion of patients with peripheral arterial occlusive disease (*n* = 19, 38%; *p* = 0.01).

All other pre-existing conditions listed here did not show statistically significant differences between the groups and can be obtained from the tables.

The proportion of patients with peripheral arterial occlusive disease in the “prone” group was 38%. In comparison with the “supine” group, this difference was statistically significant (*p* = 0.01).

### 4.3. Risk Factors

Table 6 presents the preoperative risk factors for coronary artery disease in both study groups.

Arterial hypertension was the predominant risk factor, present in 88% of patients in the “supine” group, followed by hypercholesterolemia (76%) and obesity (76%).

The “prone” group also showed arterial hypertension in most patients (86%), followed by hypercholesterolemia (78%) and obesity (70%).

### 4.4. Intraoperative Data

A single bypass procedure was performed only in the “supine” group in one patient (2%). Double bypass grafting was used more frequently in the “prone” group compared to the “supine” group (“prone”: *n* = 9/18%; “supine”: *n* = 2/4%). Triple bypass grafting was also more common in the “prone” group (“prone”: *n* = 23/46%; “supine”: *n* = 9/18%). In contrast, quadruple (*n* = 17/34%) and quintuple (*n* = 12/24%) bypass procedures were more frequently performed in the “supine” group. More than five bypass grafts were implanted in 9 patients (18%) in the “supine” group, whereas this was observed in only one patient (2%) in the “prone” group. In the “supine” group, fourfold and more than fivefold bypass grafts were performed more frequently compared to the “prone” group. In contrast, triple bypass grafting was more commonly used in the “prone” group.

The radial artery (RA) was used as a graft in all patients and was combined with an additional arterial conduit (LIMA or RIMA) and, in some cases, with the great saphenous vein (GSV).

**Figure 1:**
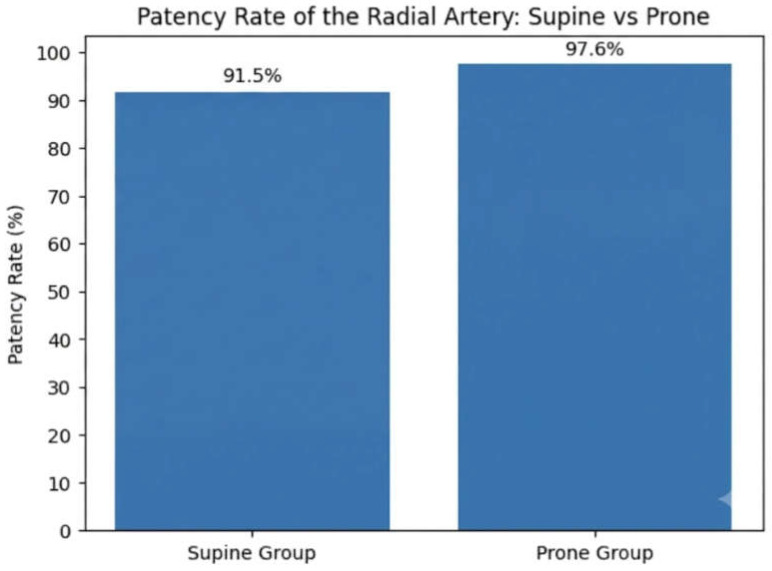
Patency rate of the RA “Supine” (*n* = 50) vs. “prone” (*n* = 50).

The distribution of graft configurations in the “prone” (*n* = 50) and “supine” (*n* = 50) groups is shown in Figure 1. In the “supine” group, the most frequently used combination was RA, LIMA, and RIMA (*n* = 20/40%), whereas in the “prone” group, the combination of RA and LIMA was more common (*n* = 19/38%). In the “supine” group, the combination of RA, LIMA, and RIMA was used more frequently. In contrast, in the “prone” group, bypass grafting was more commonly performed using the RA and LIMA combination.

### 4.5. Radial Artery (RA)

The radial artery (RA) was used as a graft in all patients in both study groups (*n* = 100/100%). In the “prone” group, the RA was positioned in a prone configuration relative to the heart (*n* = 50/50%), whereas in the “supine” group, it was positioned in a supine configuration (*n* = 50/50%).

The proximal anastomosis of the radial artery was performed in the “supine” group in 98% of patients (*n* = 49) with the ascending aorta and in 2% (*n* = 1) with the left internal mammary artery (LIMA). In the “prone” group, the proximal anastomosis was also most frequently created with the ascending aorta (90%, *n* =45), while in 10% of cases (*n* = 5) the LIMA was used.

### 4.6. Follow-Up

Clinical follow-up was obtained at a mean of 4.25 years after surgery using a standardized questionnaire. Overall clinical outcomes were favorable in both groups. No postoperative myocardial infarction and no repeat surgical reoperation were reported during follow-up. Symptoms such as dyspnea and angina pectoris were infrequent overall, although recurrent symptoms were numerically more common in the prone group.

### 4.7. Questionnaire

The medical history data obtained via telephone interview prior to MDCT imaging in the “prone” (*n* = 50) and “supine” (*n* = 50) groups are presented in this section. The most relevant findings are summarized in Table 7. These include the patients’ current health status at the time of the interview, postoperative reinterventions, and reassessment of cardiovascular risk factors.

During follow-up, catheter-based coronary re-intervention was reported in 3 patients (6%) in the supine group and in 8 patients (16%) in the prone group. However, the present analysis was not designed to attribute repeat intervention to a specific graft. Therefore, these procedures cannot be interpreted as evidence of radial artery graft failure alone, but may also reflect progression of native coronary artery disease, disease in other grafted coronary territories, or a higher overall burden of systemic atherosclerotic disease.

### 4.8. Patency Rates of the IMA and GSV

Considering the entire study population (*n* = 100), the internal mammary artery (IMA) (*n* = 98) demonstrated a patency rate of 92.84%.

Among the evaluated patients, the great saphenous vein (GSV) was used in 31 cases and showed a patency rate of 96.43%.

### 4.9. Patency Rate of the Radial Artery

The radial artery was used in all patients included in this study (*n* = 100). All results are presented in the following section comparing the “prone” (*n* = 50) and “supine” (*n* = 50) groups.

The overall patency rate of the study population (*n* = 100) was 94%.

Figure 1 illustrates the patency rates of the two groups, “prone” versus “supine,” which showed no statistically significant difference (*p* = 0.37).

MDCT-based graft patency analysis was available in 89 of the 100 patients included in the clinical follow-up cohort. Among these patients, the overall patency rate of the radial artery graft was 94%. Patency of the radial artery graft to the right coronary artery was 91.5% in the supine group and 97.6% in the prone group, without a statistically significant difference between the two graft orientations (*p* = 0.37).

For contextual comparison, the patency rate of internal mammary artery grafts was 92.8%, while the patency rate of great saphenous vein grafts was 96.4% in the evaluated subgroup. Competitive flow showed a near-significant association with radial artery graft occlusion (*p* = 0.09).

## 5. Discussion

Since its reintroduction in the 1990s, the radial artery (RA) has become a cornerstone in multi-arterial grafting (MAG) strategies aimed at achieving complete arterial myocardial revascularization [4]. Modern clinical evidence overwhelmingly supports the RA as a highly durable conduit. Current European society guidelines recommend the RA as the preferred second conduit over the saphenous vein graft (SVG) to maximize long-term patency and minimize major adverse cardiac events (MACE) [5].

The present study evaluated the mid-term patency of pedicled radial artery grafts to the right coronary artery as a function of fascial orientation. The principal finding of this retrospective cohort analysis is that both radial artery orientations were associated with excellent graft patency at a mean follow-up of 4.25 years, without a statistically significant difference between the supine and prone groups. The mid-term patency rate of the RA was 94%, which strongly aligns with the 10-year results of the landmark RAPCO randomized trial, which demonstrated the definitive long-term non-inferiority of the RA compared to the right internal mammary artery (RIMA) [6]. While historic concerns focused on an increased tendency toward vasospasm, advanced atraumatic harvesting techniques and standard pharmacological protocols have minimized this risk, establishing the RA as a durable and reliable option [7,8].

A persistent clinical and anatomical challenge is the historically reported lower patency of the RA when anastomosed to the right coronary artery (RCA) territory [9]. This structural reduction has been attributed to the specific routing required around the acute margin of the heart, exposing the graft to substantial mechanical tension and subsequent kinking [10].

To address this mechanical vulnerability, our study investigated the impact of graft orientation relative to the heart, specifically focusing on a pedicled RA to the distal RCA. We evaluated two groups defined by the spatial orientation of the fascial layer (“supine” vs. “prone” relative to the epicardium) using non-invasive cardiac CT angiography.

Clinically, our data demonstrated no statistically significant difference in patency rates between the two positioning groups. This indicates that preserving the structural integrity of the pedicled graft, utilizing the harvesting technique with accompanying veins, surrounding adipose tissue, and adventitia, provides critical baseline stability that effectively mitigates the expected negative impact of the spatial orientation around the acute margin [11].

### 5.1. Factors Influencing Graft Patency and Long-Term Survival

Graft occlusion remains a multifactorial process, with competitive flow acting as the primary hemodynamic determinant of RA failure. Contemporary data from the STS database confirm that a target vessel stenosis of less than 70% to 75% significantly increases the risk of RA graft occlusion due to competitive flow from the native coronary circulation [12]. This factor may also explain historic discrepancies in RCA patency, where moderate target vessel stenoses are clinically common.

Regarding patient-specific baselines, our study found that traditional cardiovascular risk factors did not significantly alter graft durability. However, a body mass index (BMI) greater than 25 kg/m^2^ was significantly associated with higher occlusion rates (*p* = 0.04), highlighting the negative impact of metabolic syndrome components on long-term bypass durability [13].

Furthermore, recent findings from the ongoing global ROMA trial show that the RA provides powerful long-term protective benefits against graft failure across diverse patient populations, emphasizing its survival benefit in both male and female cohorts compared to conventional vein grafts [14].

From a clinical perspective, the findings remain relevant. The data indicate that pedicled radial artery grafting to the right coronary artery can achieve excellent mid-term patency in both fascial orientations. This may be reassuring for surgeons when graft orientation is determined by intraoperative anatomy, conduit length, lie of the graft, or technical feasibility rather than by a presumed patency advantage of one orientation over the other.

### 5.2. Study Limitations

The findings of this study should be interpreted within the context of its retrospective, non-randomized design. Graft orientation reflected the standardized operative technique of the respective surgeon and was not protocol-assigned. Nevertheless, the homogeneous study population, uniform surgical strategy, and objective MDCT-based assessment of graft patency strengthen the validity of the present observations.

## 6. Conclusions

Pedicled radial artery grafts to the right coronary artery demonstrated excellent mid-term patency. In this cohort, the spatial orientation of the pedicled radial artery graft, with the fascial side facing either the sternum or the epicardium, was not associated with a significant difference in graft patency. These findings suggest that graft orientation may be determined according to intraoperative anatomical and technical considerations without compromising mid-term graft patency.

## Figures and Tables

**Table 1:** Preoperative variables.

Category	Variables
Demographics	Age, sex, body mass index (BMI)
Clinical status	CCS class (I–IV), NYHA class (I–IV)
Cardiac history	Myocardial infarction (including < 90 days), supraventricular arrhythmias
Coronary interventions	PCI/stenting (LAD, LCX, RCA)
Risk factors	Hypertension, hypercholesterolemia, diabetes mellitus (IDDM/NIDDM), COPD, peripheral arterial disease, cerebrovascular disease, chronic kidney disease
Coronary anatomy	1-, 2-, 3-vessel disease, left main disease
Cardiac function	Left ventricular ejection fraction (categorical and mean EF)
Risk score	EuroSCORE

**Table 2:** Intraoperative variables.

Category	Variables
Surgical times	Operation time, cardiopulmonary bypass time, aortic cross-clamp time
Technique	On-pump CABG, type of cardioplegia
Grafts	LIMA, RIMA, radial artery, great saphenous vein combinations
Anastomoses	Number of distal anastomoses (1–5, >5), completeness of revascularization
Technical parameters	Target vessel diameter (>1.5 mm), proximal radial artery anastomosis site (ascending aorta or LIMA)
Study variable	Radial artery orientation (supine vs. prone)
Support	Inotropic medication use

**Table 3:** Postoperative outcomes.

Category	Variables
ICU/ventilation	ICU stay, mechanical ventilation > 24 h
Cardiac markers	Peak CK-MB, myocardial infarction (CK-MB > 80 U/L)
Rhythm	Atrial fibrillation
Blood products	RBC, FFP, platelet transfusion
Wound complications	Sternal wound infection, limb complications (infection, bleeding, paresthesia, dysfunction)
Reinterventions	Reoperation for bleeding, angiography, PCI, graft failure surgery
Devices	Intra-aortic balloon pump (IABP)
Major outcomes	Stroke, mortality

**Table 4:** Demographic data of the “supine” patient group (*n* = 50) and “prone” patient group (*n* = 50).

Parameter	Supine Group (*n* = 50)(Mean ± SD/Median [Min–Max])	Prone Group (*n* = 50)(Mean ± SD/Median [Min–Max])	*p*-Value
Age (years)	64.1 ± 8.28 66 (46–78)	62.16 ± 10.00 61 (41–79)	0.276
BMI (kg/m^2^)	28.01 ± 3.71 27.78 (20.97–37.76)	28.58 ± 4.62 27.90 (20.08–38.37)	0.495

**Table 5:** Medical history data of the “supine” patient group (*n* = 50) and the “prone” patient group (*n* = 50).

Variable	Supine Group (*n* = 50) *n* (%)	Prone Group (*n* = 50) *n* (%)	*p*-Value
History of myocardial infarction	13 (26%)	17 (34%)	0.383
Myocardial infarction ≤ 90 days	4 (8%)	6 (12%)	0.739
Supraventricular arrhythmias	7 (14%)	10 (20%)	0.417
Peripheral arterial occlusive disease	7 (14%)	19 (38%)	0.006
Cerebrovascular disease	3 (6%)	5 (10%)	0.712
COPD	2 (4%)	5 (10%)	0.429
Chronic renal insufficiency	4 (8%)	–	–

**Table 6:** Risk factors in the “supine” patient group (*n* = 50) and the “prone” patient group (*n* = 50).

Variable	Supine Group (*n* = 50) *n* (%)	Prone Group (*n* = 50) *n* (%)	*p*-Value
Arterial hypertension	44 (88%)	43 (86%)	0.766
Hypercholesterolemia	38 (76%)	39 (78%)	0.81
Obesity (BMI > 25 kg/m^2^)	38 (76%)	35 (70%)	0.499
Obesity (BMI > 30 kg/m^2^)	12 (24%)	15 (30%)	0.501
Insulin-dependent diabetes mellitus	4 (8%)	7 (14%)	0.518
Non-insulin-dependent diabetes mellitus	9 (18%)	6 (12%)	0.401
Nicotine abuse	3 (6%)	4 (8%)	1

**Table 7:** Medical History Data Obtained by Questionnaire in the Investigated “Supine” Patient Group (*n* = 50) and “Prone” Patient Group (*n* = 50).

Medical History Data	Supine Group (*n* = 50) *n* (%)	Prone Group (*n* = 50) *n* (%)	*p*-Value
**Health Status**			
Myocardial infarction (postoperative)	0 (0%)	0 (0%)	1
Dyspnea on severe exertion	8 (16%)	11 (22%)	0.611
Dyspnea on mild exertion	6 (12%)	4 (8%)	0.739
Dyspnea at rest	2 (4%)	0 (0%)	0.495
Angina on severe exertion	1 (2%)	2 (4%)	1
Mild symptoms during normal exertion	0 (0%)	0 (0%)	1
Severe symptoms during normal exertion	0 (0%)	1 (2%)	1
Angina at rest	1 (2%)	3 (6%)	0.617
Hypoesthesia/Paresthesia	13 (26%)	19 (38%)	0.197
Thenar hypotrophy	1 (2%)	0 (0%)	1
**Postoperative Re-interventions**			
PTCA with stent/balloon dilatation	3 (6%)	8 (16%)	0.2
Re-operation	0 (0%)	0 (0%)	1
**Risk Factors**			
Diabetes mellitus	15 (30%)	15 (30%)	1
Arterial hypertension	48 (96%)	44 (88%)	0.269
Renal insufficiency	5 (10%)	5 (10%)	1
Hypercholesterolemia	42 (84%)	40 (80%)	0.795
Smoking	3 (6%)	4 (8%)	1

## Data Availability

The data presented in this study are available on request from the corresponding author (the data are not publicly available due to privacy or ethical restrictions).

## List of Abbreviations

BIAS	Biometric analysis of samples
BMI	Body mass index
CABG	Coronary artery bypass grafting
CCS	Canadian Cardiovascular Society
COPD	Chronic obstructive pulmonary disease
EACTS	European Association for Cardio-Thoracic Surgery
FFP	Fresh frozen plasma
GSV	Great saphenous vein
IABP	Intra-aortic balloon pump
ICU	Intensive care unit
IDDM	Insulin-dependent diabetes mellitus
IMA	Internal mammary artery
LAD	Left anterior descending artery
LCX	Left circumflex artery
LIMA	Left internal mammary artery
MACE	Major adverse cardiac events
MAG	Multi-arterial grafting
MDCT	Multidetector computed tomography
NIDDM	Non-insulin-dependent diabetes mellitus
NYHA	New York Heart Association
PCI	Percutaneous coronary intervention
PTCA	Percutaneous transluminal coronary angioplasty
RA	Radial artery
RAPCO	Radial Artery Patency and Clinical Outcomes trial
RBC	Red blood cells
RCA	Right coronary artery
RIMA	Right internal mammary artery
ROMA	Randomized comparison of the clinical Outcome of single versus Multiple Arterial grafts
STS	Society of Thoracic Surgeons
SVG	Saphenous vein graft

## References

[1] Gaudino M., Bakaeen F.G., Sandner S., Aldea G.S., Arai H., Chikwe J., Firestone S., Fremes S.E., Gomes W.J., Bong-Kim K. (2023). Expert systematic review on the choice of conduits for coronary artery bypass grafting: Endorsed by the European Association for Cardio-Thoracic Surgery (EACTS) and The Society of Thoracic Surgeons (STS). J. Thorac. Cardiovasc. Surg..

[2] Dimagli A., Soletti G., Harik L., Perezgrovas Olaria R., Cancelli G., An K.R., Alzghari T., Mack C., Gaudino M. (2023). Angiographic Outcomes for Arterial and Venous Conduits Used in CABG. J. Clin. Med..

[3] Dashwood M.R., Celik Z., Topal G. (2025). Reducing vasospasm of vein and arterial conduits used in coronary artery bypass surgery: Are solutions the solution or is preserved perivascular fat the answer?. Front. Physiol..

[4] Gaudino M., Chikwe J., Falk V., Lawton J.S., Puskas J.D., Taggart D.P. (2020). Transatlantic Editorial: The Use of Multiple Arterial Grafts for Coronary Revascularization in Europe and North America. Ann. Thorac. Surg..

[5] Neumann F.J., Sousa-Uva M. (2019). ‘Ten commandments’ for the 2018 ESC/EACTS Guidelines on Myocardial Revascularization. Eur. Heart J..

[6] Buxton B.F., Hayward P.A., Raman J., Moten S.C., Rosalion A., Gordon I., Seevanayagam S., Matalanis G., Benedetto U., Gaudino M. (2020). Long-Term Results of the RAPCO Trials. Circulation.

[7] Achouh P., Isselmou K.O., Boutekadjirt R., D’Alessandro C., Pagny J.Y., Fouquet R., Fabiani J.N., Acar C. (2012). Reappraisal of a 20-year experience with the radial artery as a conduit for coronary bypass grafting. Eur. J. Cardiothorac. Surg..

[8] Lawton J.S., Tamis-Holland J.E., Bangalore S., Bates E.R., Beckie T.M., Bischoff J.M., Bittl J.A., Cohen M.G., DiMaio J.M., Don C.W. (2022). 2021 ACC/AHA/SCAI Guideline for Coronary Artery Revascularization: A Report of the American College of Cardiology/American Heart Association Joint Committee on Clinical Practice Guidelines. Circulation.

[9] Tatoulis J., Buxton B.F., Fuller J.A., Meswani M., Theodore S., Powar N., Wynne R. (2009). Long-term patency of 1108 radial arterial-coronary angiograms over 10 years. Ann. Thorac. Surg..

[10] Gaudino M., Fremes S., Schwann T.A., Tatoulis J., Wingo M., Tranbaugh R.F. (2019). Technical Aspects of the Use of the Radial Artery in Coronary Artery Bypass Surgery. Ann. Thorac. Surg..

[11] Blitz A., Osterday R.M., Brodman R.F. (2013). Harvesting the radial artery. Ann. Cardiothorac. Surg..

[12] Nappi F., Bellomo F., Nappi P., Chello C., Iervolino A., Chello M., Acar C. (2021). The Use of Radial Artery for CABG: An Update. Biomed. Res. Int..

[13] Singh S.K., Desai N.D., Petroff S.D., Deb S., Cohen E.A., Radhakrishnan S., Schwartz L., Dubbin J., Fremes S.E. (2008). Radial Artery Patency Study Investigators. The impact of diabetic status on coronary artery bypass graft patency: Insights from the radial artery patency study. Circulation.

[14] Gaudino M., Benedetto U., Fremes S., Biondi-Zoccai G., Sedrakyan A., Puskas J.D., Angelini G.D., Buxton B., Frati G., Hare D.L. (2018). Radial-Artery or Saphenous-Vein Grafts in Coronary-Artery Bypass Surgery. N. Engl. J. Med..

